# Collection and Processing of Data from Wrist Wearable Devices in Heterogeneous and Multiple-User Scenarios

**DOI:** 10.3390/s16091538

**Published:** 2016-09-21

**Authors:** Francisco de Arriba-Pérez, Manuel Caeiro-Rodríguez, Juan M. Santos-Gago

**Affiliations:** Department of Telematics Engineering, University of Vigo, Campus Lagoas-Marcosende, Vigo 36310, Spain; Manuel.Caeiro@det.uvigo.es (M.C.-R.); Juan.Santos@det.uvigo.es (J.M.S.-G.)

**Keywords:** wearable sensors, wearable computing, data interoperability, internet of things, machine to machine

## Abstract

Over recent years, we have witnessed the development of mobile and wearable technologies to collect data from human vital signs and activities. Nowadays, wrist wearables including sensors (e.g., heart rate, accelerometer, pedometer) that provide valuable data are common in market. We are working on the analytic exploitation of this kind of data towards the support of learners and teachers in educational contexts. More precisely, sleep and stress indicators are defined to assist teachers and learners on the regulation of their activities. During this development, we have identified interoperability challenges related to the collection and processing of data from wearable devices. Different vendors adopt specific approaches about the way data can be collected from wearables into third-party systems. This hinders such developments as the one that we are carrying out. This paper contributes to identifying key interoperability issues in this kind of scenario and proposes guidelines to solve them. Taking into account these topics, this work is situated in the context of the standardization activities being carried out in the Internet of Things and Machine to Machine domains.

## 1. Introduction

In recent years, the consumer electronics industry has made a huge investment in wearable technology. Companies are producing many different wearable devices: fitness trackers, smart watches, connected headsets, smart glasses, wrist bands, etc. Despite wearables not being new, the development of mobile technologies and the quantified-self movement related to fitness and sport activities have led to their explosion [1,2]. Among the wide variety of wearable devices, wrist wearables such as smartwatches and wrist bands seem to have become mainstream. Estimations indicate that by 2019 [3] wrist wearables will reach 100 million sold units, while all the other wearable devices together will achieve just 7.3 million units. In addition to other features related to their reduced size and comfortable use, wrist wearables include a good amount of sensors providing continuous data about vital signs (e.g., heart rate, skin temperature) and environmental variables (e.g., movements) that can be used for many different purposes.

Similarly to the market development, many references to the use of wearables can be found in recent scientific literature, not only as wrist devices, but also as tattoos, clothes worn or diapers [4]. For example, LilyPad is a processor to support the development of intelligent clothes that has been used to prototype new products [5,6]. The use of these wearables, especially wrist devices, is being adopted to carry out research focused on the detection of human features, including issues such as sleep and stress as well as analysing human activities such as writing, drinking coffee, giving a talk, etc. [7,8,9,10,11,12]. These initiatives involve different options to perform the analytics of wearable sensors’ data.

In our case, following the trends in the market and scientific domain, we are taking advantage of these new devices to develop solutions for teachers and students in educational contexts. Particularly, we are developing sleep and stress indicators gathered from students [13,14,15], in a similar way to how it is being explored by recent research projects [16,17,18]. This information can be used to develop a better knowledge about the learner engagement, to identify the preferred activities for students, to discover learning difficulties or to promote the development of good habits. A key requirement in our piece of research is to be able to work with data coming from the wearable devices of different vendors. This scenario introduces many interoperability issues because nowadays different wearable devices include different sensors, involve specific ways to collect data, provide data in accordance to different data models, etc. We need to take into account all these differences and develop appropriate interoperability solutions. This work is of great interest, because collecting and integrating data from multiple sources is a situation that can be produced in many different scenarios.

These issues are relevant in the context of the standardization activities in the Internet of Things [19] and Machine to Machine domains [20] domains. There exists recent literature about the interoperability of devices in different environments [21,22,23] and initiatives to define standards, such as: ISO/IEC JTC1 SWG5 and WG10; IEEE “Standard for an Architectural Framework for the Internet of Things” [24]; oneM2M Standards for M2M and the Internet of Things [25] “Functional Architecture” [26]; etc. Furthermore, this piece of research is situated in the new area of multimodal learning analytic, which takes into account not just learners’ logs on learning systems, but also audio, video, location, motion, temperature, humidity or luminosity data, among others [27]. The use of wearables in learning contexts is being explored in other pieces of research, for example to support learners as makers [28].

This paper introduces and analyses the interoperability issues involved in wearable scenarios: the variety of devices, sensors and operating systems available, the different options to transfer data from wearables to third-party servers, and issues related to the data models. A review of the main software integration platforms including Google Fit/Android Wear, and iOS, is provided. We also analyse solutions offered by some vendors, more based on a specific device than on the provision of an integrated platform for several devices, such as Fitbit, Microsoft, and Jawbone. Finally, we show our experience and results using the following wrist wearables: Fitbit Surge, LG Watch R, Microsoft Band and Jawbone UP Move. In the second part of the paper our use cases are introduced. We describe our experience with a set of wearables to calculate sleep and stress indicators for educational purposes. We also show the architecture developed and explain the interoperability issues related to data access, data models, etc.

The paper is organized as follows: The next section introduces a review of the fragmentation problem in the wrist wearable market and reviews the current state of wearables, platforms and sensors. This section has been prepared as a personal compilation using data from recognized sources such as IDC (International Data Corporation) Research Inc., wearable vendors, etc. Then, Section 3 analyses the different options available to collect data from wearables towards third-party systems and Section 4 discusses differences in data models. The purpose of Section 3 and Section 4 is to show all the identified problems related to the collection and processing of wearable data. Next, Section 5 introduces our experience on the development of wearable-based solutions and their application to educational contexts, considering the devices, data transfer and analytic processing. Section 6 describes the standardization context in which this work is being performed. The paper finishes with conclusions in Section 7.

## 2. Fragmentation Problems

The fragmentation problem is well-known in the smartphone domain, especially related to the Android operating system [29]. The diversity of vendors, screen sizes and versions have produced a large variety of devices and configurations. In a similar way, this problem is being replicated in wearable devices.

### 2.1. Devices

**Figure 1:**
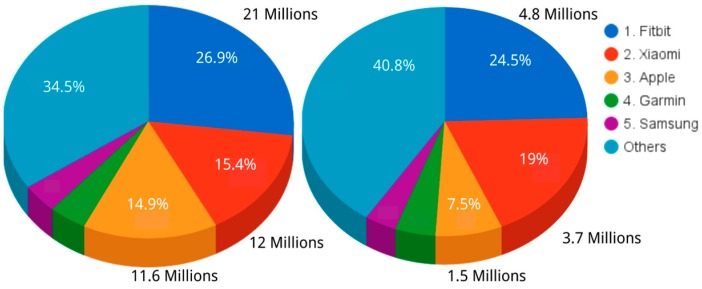
Wrist wearable devices’ 2015 market share on the left side, 1Q2016 market share on the right side, both by IDC Research Inc. (Framingham, MA, USA) (Adapted from [30,31]).

Nowadays, there are many different vendors of wearable devices. The left side of Figure 1 shows the market share of the main devices based on data from the whole 2015 year [30]. Fitbit was the leading company in the sector of smartbands as it covered more than one third of the market share. Next, developers were Xiaomi and Apple, with around 15% of the market share each. It is important to notice that with a market share under 5% there were more than 40% of devices from several companies. In 2015, a total number of 78.8 million units of wrist wearables were sold worldwide. This trend continues in 2016, because in the first quarter of the year around 20 million units have already been sold [31]. Figure 1 on the right side shows similar figures to the 2015 ones during the first quarter of 2016. These figures show that wrist wearables are being consolidated as a new electronic consumer product, whose adoption is predicted to increase over the next year [32].

### 2.2. OSs and Software Platforms

Wrist wearable devices are being developed in a variety of ways with differences in shape, purpose, hardware features, etc. It is possible to classify them into two categories attending to their functionalities. On the one hand, there are simple devices with limited capabilities, named as smartbands, whose purpose is to quantify very specific features such as user physical activity, sleep, etc. On the other hand, there are more advanced and general purpose devices, known as smartwatches. They include an embedded OS that enables the installation of third-party applications and functionalities similar to the ones available in smartphones. This classification is key to carry out any development or research in this domain, because the chances to collect data are very different. In general, smartbands do not facilitate solutions to transfer data to third-party systems.

Taking into account market shares for both (quantifying) smartbands and smartwatches [31], nowadays, the number of smartbands is much larger than smartwatches. Also, the smartbands market is very fragmented, more than 40% of devices have a market share of less than 5%. In the smartwatch market the fragmentation is smaller. Apple leads the smartwatch market with a share of more than 45%, followed by Samsung with more than 20% and Motorola with a 10% share.

**Figure 2:**
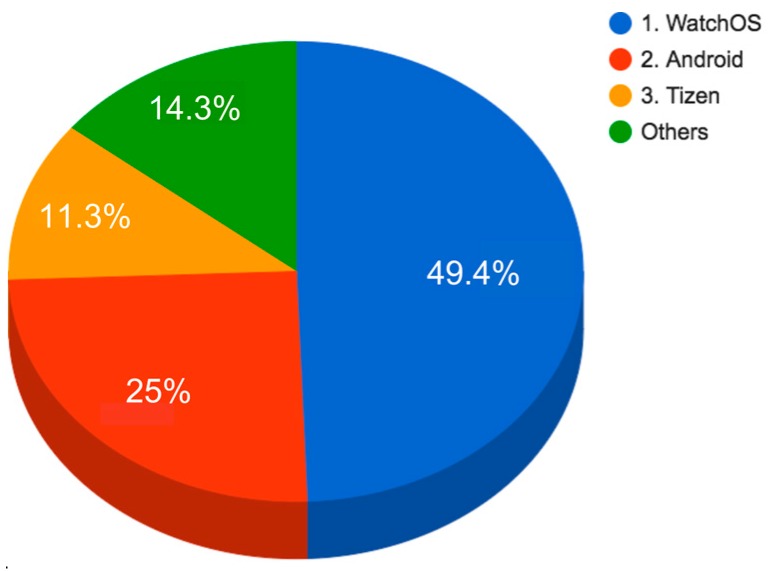
2016 smartwatch OS prediction in the wrist wearable sector by IDC Research Inc. Adapted from [32].

In the smartwatches domain it is very relevant to analyse the OSs and software platforms. Smartwatches need an OS to run apps and services. IDC studies about current systems and 2016 predictions show the Apple Watch OS with almost 50% of the market share, followed in second place by Android with almost 25%, and in third place Samsung Tizen with 11.3%, (cf. Figure 2). These companies have developed software platforms involving several systems (apps, dashboards, SDKs (Software Development Kits) and REST APIs (RE presentational State Transfer Application Programming Interfaces) to support final users and third-party developers. Each platform enables the collection of data from devices of the same company and partners. Using services available on these platforms, final users can gain access to their own collected data and third-party developers can build new applications and services making use of the collected data under certain conditions. Next, the main platforms are described:

- **Apple Health** [33]. The Apple Health platform enables the collection of data from devices and apps of Apple, but it also supports some other companies, such as Nike, Fitbit and RunKeeper. It allows the user to view data and information related to physical activities and health. Two different kinds of profiles are recognized by Apple Health: general purpose and medical research. For medical research purposes, Apple Health provides more data analysis capabilities than the general purpose profile [34].
- **Google Fit + Android Wear** [35]. Google Fit platform supports the collection of data from devices and apps under the Android Wear, Android and associated partners. Indeed, Google Fit supports more partners than any other platform [35], such as: Nike, Adidas, Xiaomi, HTC, Motorola, LG. Final users can view the information collected in the Google Fit web or in the app dashboard.
- **S-Heath** [36]. This is the Samsung platform supporting all the wearables under its Tizen OS. It also works with devices delivered with the Android Wear, and devices and apps of some partners, such as Nike and Sleep as Android. Originally, it was proposed as a unifying platform for Samsung devices, but now it is open to other platforms. In any case, this platform has the smaller number of associated partners and market share, mainly located in the Asia area.

In addition to the main platforms, there exist other ones supporting specific devices such as Microsoft, Fitbit or Jawbone. Nevertheless, these platforms are oriented to store and provide data collected from the specific device. To the best of our knowledge, to date, they have not been focused on supporting a homogeneous solution to integrate data from wearables of other vendors.

### 2.3. Sensors

**Figure 3:**
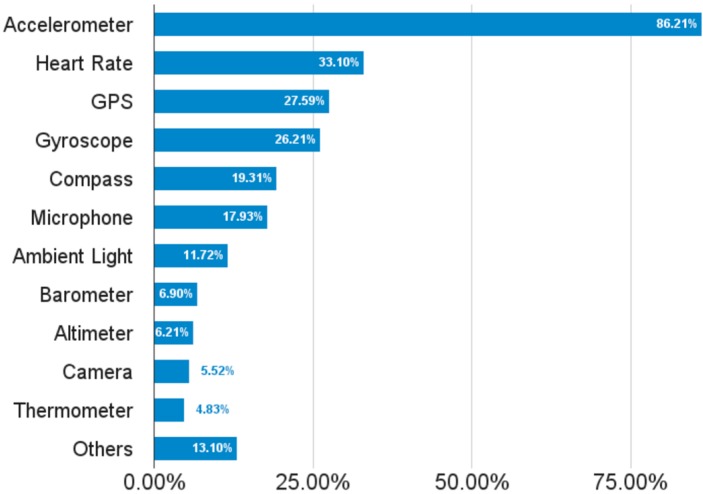
Sensors available in wearables.

In addition to the variety of devices, OSs and platforms, there is also a great variety in sensors included in wearables. Figure 3 shows the percentage of sensors available in the more than 140 wrist wearables analyzed in our piece of research [37,38,39,40,41,42,43,44,45,46]. As is shown in the figure, the Heart Rate (HR) and movement-related sensors (e.g., accelerometer, GPS, gyroscope, compass) are the most common ones. The variety of sensors provides a new source of diversity. Moreover, there are many differences related to the precision of sensors [47,48,49,50], that can depend on how the wearable is situated [51,52]. In any case, it is generally accepted that sensors’ precision is enough for non-medical purposes, such as self-quantification and fitness scenarios. As our piece of research is situated in this context, we do not tackle these issues in this paper.

## 3. Data Collection

Wearables allow different ways in which data from sensors can be collected by other systems. In this section, we introduce a technical description to show the different systems involved and options available.

### 3.1. Involved Systems

**Figure 4:**
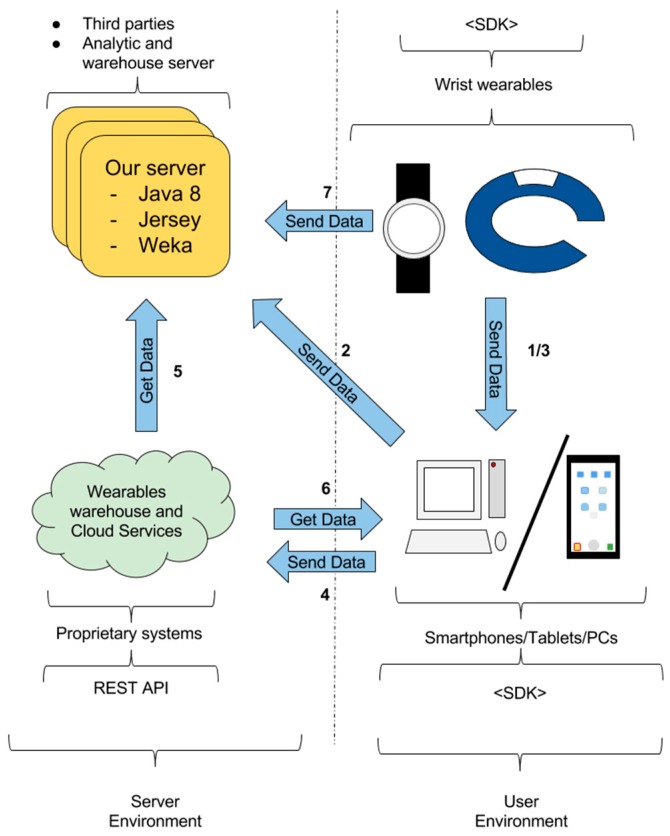
Systems involved in the smartwatch data collection scenario.

Figure 4 shows the several systems that can be involved in the smartwatch data collection scenario: wearables, smartphones, computers and servers/cloud services. Two types of systems are distinguished: proprietary and third-party. Proprietary systems can be found as wearable devices, apps for smartphones and computers, and cloud services. These systems are provided and maintained by wearable vendors to collect users’ data, to perform analytics and to provide data and analytic results to users and to authorized third-parties. Third-party systems such as services, apps for smartphones and wearables, and programs for computers can be developed and maintained by external entities to provide specific functionalities. Each one of these components is intended to perform some specific functions:

- Data collection. Data is captured by the sensors available in wearable devices and smartphones.
- Data transfer. Data collected in wearables can be transferred to a computer or to a smartphone as an intermediate step towards its eventual transfer to a permanent data storage. This transfer can be produced through proprietary solutions or using third-party apps and programs. In theory, wearable data could be directly transferred from the wearable to the permanent data storage, without the need of any intermediate system, but nowadays, this is not possible.
- Permanent data storage. This function is related to the permanent storage of the data. Usually, this permanent storage is performed in proprietary servers, where third parties and final users can gain access to the data.
- Data analysis. This is related to the analytic processing of data to provide results of interest. Typically, this processing is performed in servers, both proprietary and third-party.

In order to perform the transfer of data to the third-party server, several options can be available depending on the wearable. Check Figure 4 clock-wise. In some cases, wearable device vendors (e.g., iOS) provide SDKs to enable the development of apps for wearables to directly collect and send data. In the case of smartphones, tablets and PCs, SDKs are available to develop apps that collect data directly from linked smartwatches, from the smartphone itself or from a proprietary warehouse. Using these SDKs, third-parties can develop specific software to collect data on wearables and send it to other systems. Proprietary warehouses and cloud services usually provide a REST API. This REST API allows third-parties to gain access to users’ data.

The main smartwatch platforms include the following systems:

- **Apple Health.** Apple Health is made up of a warehouse and cloud services, an app, and a SDK (Health Kit). The Apple Health warehouse and cloud services maintain all the data collected from users and provide some analytic services. Nevertheless, Apple Health does not provide any REST API for third-party systems. The app is used in the iPhone or iPad to synchronize all the available data with the Apple Health server and to show such data to the user. The Health Kit SDK enables the development of apps for iWatch, iPhone and iPad providing access to data available in the Apple Health warehouse.
- **Google Fit.** It is the most complete solution, including a warehouse and cloud services and an app for Android systems. It provides a SDK for app developers, a SDK to gain access to Google Fit warehouse and a warehouse REST API for third-party systems. The Android app is needed to transfer the data from the wearable to the smartphone and then to the proprietary warehouse. This app involves some intelligence because it selects the best sensors available to collect data depending on the circumstances, whether they are in the wearable or in the smartphone. For example, if we are walking, it will use the pedometer to count the number of steps. Nevertheless, if we go by bike, as movement in the wrist will be limited, this app will measure the distance based on data from the smartphone GPS sensor. Eventually, measures of distance, steps, type and time of physical activity are calculated using the best data available.
- **S-Health.** Similarly, a smartphone app is included to synchronize the data with the Samsung server. It also provides a SDK to support the development of apps by third-party developers, gaining access to collected data in the proprietary warehouse (SDK-Warehouse). Nevertheless, this SDK does not enable the access to wearable data sensors directly. The S-Health platform does not include any REST API.

Note that not all the systems available are included in these three main platforms. In addition, beyond them, there exist other developers providing their own solutions involving REST APIs and SDKs. Some examples:

- Fitbit. It only provides a warehouse REST API for third-party systems. This platform synchronizes data from Fitbit quantification bands and enables third-party developers to get such data through the REST API. For some data, such as minute by minute heart rate, third-party developers need to own a specific permission.
- Microsoft Health. It provides a SDK for app developers and a REST API for third-party systems. Using these two facilities it is possible to gain access to data available in the Microsoft cloud that has been collected from Microsoft Band devices.
- Jawbone. It provides a SDK to support the development of apps by third-party developers gaining access to collected data in the proprietary warehouse (SDK-Warehouse) and a warehouse REST API for third-party systems. Similar to the previous cases, it also stores and provides data from the connected Jawbone devices.

A final summary of the systems available at each platform is shown in Table 1. This table distinguishes three options in columns: SDK-Sensors, if the platform provides a SDK enabling access to the sensors and the collection of data directly from the wearable; SDK-Warehouse, if the platform provides an SDK enabling the retrieval of data from the proprietary warehouse; or REST API if direct access to the warehouse is provided. Notice, the SDK-Warehouse solution is equivalent to the REST API. The difference is that it simplifies the sign in and the collection request, but it has to be done from a smartphone. Each one of the facilities included in the table is available for developers without any payment, one only needs to register on the corresponding platform.

In addition to the platform, each smartwatch includes a native SDK enabling the development of apps, but without any specific support for a sensors’ collected data transfer. Using these native SDKs it is possible to develop apps that collect data from wearables and transfer it through generic communication facilities (e.g., Bluetooth, Wi-Fi). These solutions are ad-hoc, involve high complexity and many issues, such as battery drains. Therefore, we focus the attention on the solutions provided by the companies to support data collection and transfer in an integrated way.

### 3.2. Data Transfer

Based on the previous description, two main modes in which data can be transferred from wearables to third-party servers can be identified:

- **Wearable data transfer.** Taking the data directly from the wearable sensors.
- **Warehouse data transfer.** Taking the data from the proprietary warehouse.

The warehouse data transfer has some disadvantages in relation to the wearable data transfer. The main one is related to the collection of data in real time. In the warehouse data transfer case, the transfer of data from the wearable to the proprietary warehouse is performed sporadically at random times. Sometimes, the time between transfers can be in the order of several days. On the contrary, in the case of wearable data transfers, this can be produced at specific time intervals. A second drawback of the warehouse data transfer is related to the nature of data. Usually, when data is transferred to the proprietary warehouse some kind of processing is performed (e.g., summarization). In the case of wearable data transfer, usually it is raw data. For example, in general the nominal values of the accelerometer cannot be collected from a warehouse.

In both modes, wearable and warehouse, it is possible to distinguish between two options in which access to data can be produced:

- **Direct access.** If the third-party collects the data directly from the source in which it is available, whether wearable or warehouse.
- **Indirect access.** If some kind of intermediary system, such as a smartphone or a PC, is needed as a gateway to the third-party server.

Taking into account these two transfers and accessing options, four different configurations can be distinguished, as it is explained in next sub-sections.

#### 3.2.1. Wearable Data Transfer—Indirect Access

**Figure 5:**
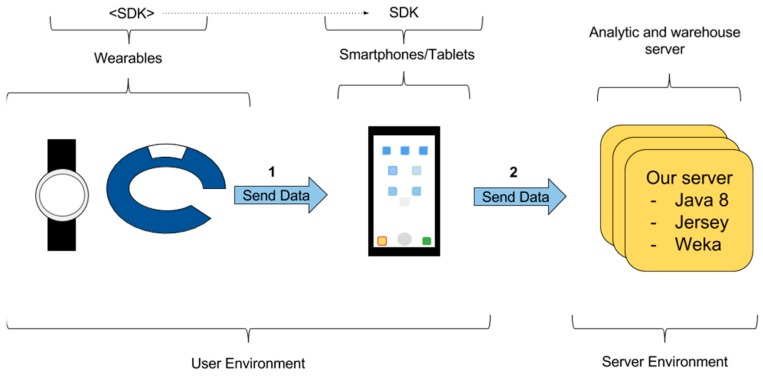
System representation for the wearable data transfer—indirect access.

The data transfer from the wearable to a third-party system can be performed using an app in an intermediate smartphone. Figure 5 shows the way in which data can be transferred through links 1 and 2:

- Link 1. An app in a smartphone can collect data from a wearable. To do so, a native app running in the smartphone subscribes as a listener to events in wearable sensors. The wearable periodically notifies new data and the smartphone collects it. In all cases where we have studied this transfer, it is performed using Bluetooth.
- Link 2. An app in a smartphone sends data to a server. Periodically, a native app running in the smartphone checks the availability of an Internet connection. When the connection is available, the app sends the data collected to the server. The smartphone app stores the data collected from the wearables while the Internet connection is not available.

This option requires a huge effort in software development. Particularly, a specific app to be executed in the smartphone needs to be developed, usually taking advantage of the SDK available. This app has to manage the subscription to events in the wearable and the issues related to possible breaks in the Internet connection. In addition, for some wearables, we also need to develop an app for the wearable to record the sensors’ data and to transfer it to the smartphone app. This is the case for Android Wear wearables.

#### 3.2.2. Warehouse Data Transfer—Direct Access

**Figure 6:**
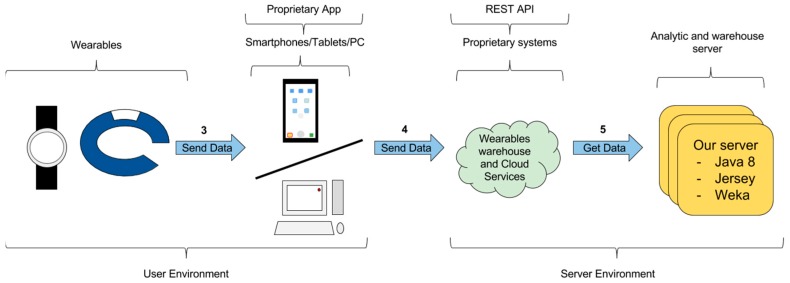
System representation for the warehouse data transfer—direct access.

The third-party server obtains the data using the proprietary warehouse REST API. Figure 6 shows the way in which data can be transferred through links 3, 4 and 5:

- Link 3. The wearable sends data to its proprietary warehouse. This is performed through the proprietary transfer solution. This solution is based on an app running in an intermediate smartphone or PC, which acts as a gateway towards the server. In the cases we studied, the wearable sends the data to the intermediate app using Bluetooth. This transfer is performed periodically because of the energy demands of the Bluetooth connection. Depending on the wearable, if this transfer cannot be performed for a long time, the data can be summarized or even lost.
- Link 4. The data is transferred from the intermediate smartphone or PC to the wearable warehouse.
- Link 5. The wearable warehouse receives requests from third-party systems through the REST API. Then, the warehouse delivers the requested data.

#### 3.2.3. Warehouse Data Transfer—Indirect Access

**Figure 7:**
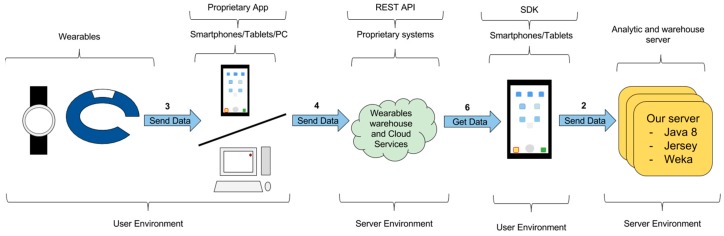
System representation for the warehouse data transfer—indirect access.

This configuration involves indirect access to the proprietary warehouse using an intermediate smartphone. Figure 7 shows the way in which data can be transferred through link 6 and link 2. From the previous configuration, an app running on the smartphone/tablet obtains the data from the proprietary warehouse and cloud service. Next, this app sends the data to the third-party server. This configuration is needed in the cases in which the warehouse does not provide a REST API but it allows operation from the SDK.

#### 3.2.4. Wearable Data Transfer—Direct Access

**Figure 8:**
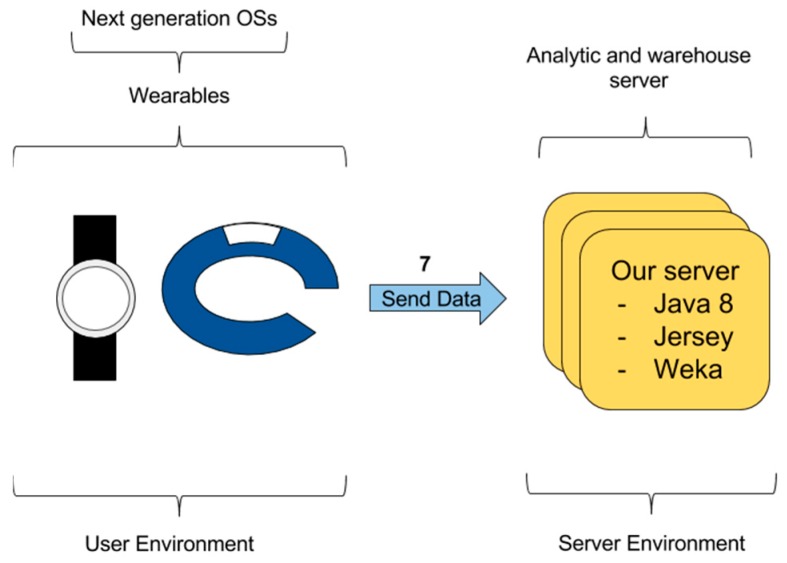
System representation for the wearable data transfer—direct access.

A wearable data transfer and direct access between the wearable and the third-party server is possible in theory, but not in practice nowadays. Figure 8 shows link 7 configuration. The intermediate app in the smartphone is removed and a direct link between the wearable and the third-party server is stablished. This could be supported by some systems, for example, new Android Wear wearables allow the inclusion of a SIM card or the use of Wi-Fi access points (e.g., Samsung Gear S2, Samsung, Seoul, Korea). Nevertheless, nowadays, this option is only available to connect the wearable to a mobile device (e.g., smartphone) and it does not support the communication to a web service. In addition, the energy consumption is too high. Therefore, this configuration is not a valid option yet.

#### 3.2.5. Data Transfer Options by Platform

The three main software platforms in the smartwatches domain support different options related to the data transfer, (cf. Table 2). In the table, we can see how each vendor supports different data transfer modes. The warehouse data transfer—direct access is the most commonly supported one.

## 4. Data Integration

This section introduces issues related to the differences among data coming from wearables of different vendors. Data such as HR, number of steps, speed or body temperature is arranged in accordance to particular vendor specifications and differences can be found at several points: data models, identification, vocabularies, availability on time, etc. As a result, it is not possible to work with data collected from different vendors directly. A homogenization process is needed to get data in accordance to the same conventions. The next sections describe these differences focusing on sleep features, because it is our research field, and these issues can be found in other fields.

### 4.1. Differences in Data Models

Data collected from wearables is arranged in temporal segments according to different models. Each model can be described in terms of the elements and attributes included as key-value pairs, as in a typical XML (eXtensible Markup Language) or JSON (JavaScript Object Notation) specification. In the case of sleep data, the following arrangements can be found:

- Microsoft manages each sleep period as a different object, named as a sleep segment. When the user moves from light sleep to deep sleep, for example, a new sleep segment object is produced. Each sleep segment includes its own start and finish date object and type of sleep (cf. Listing 1).
- Google Fit. It uses a structure based on segments similar to the Microsoft Band. Each segment includes the start and finish time and it is tagged with the sleep state.
- Apple Health. Its model is also based on segments, but in this case segments can be overlapped [30]. It is possible to find segments in which the user is in bed but not sleeping and other segments in which the user is in bed and sleeping. Situating the segments along a temporal axis, it is possible to know the different sleep states of the user, the total sleeping time, the awake time, etc.
- Fitbit. The sleep record is represented minute by minute (cf. Listing 2). Each minute is tagged with the sleep state of the user.
- In Jawbone, there are segments indicating a new sleep period, but there is no information about the end of the period (cf. Listing 3).

### 4.2. Differences in Data Names

Different vocabularies are used by wearable vendors:

- Sleep types managed by Microsoft are: Doze, Snooze, Awake, Sleep, and three subtypes: Unknown (for awake segment), RestlessSleep and RestfulSleep (for sleep segment).
- Google Fit distinguishes among four sleep levels: sleep light, deep, REM (Rapid Eye Movement) and awake. These are the exact number of states and names given by the sleep physiognomy theory.
- Fitbit distinguishes among three possible states (cf. Listing 2): sleeping, awake or really awake. The difference between awake and really awake is determined by the violence and duration of the movements. Basically, it tries to distinguish between a light-brief movement (awake) and a strong-long movement (really awake).
- Jawbone distinguishes sleep states as: awake, light and deep (cf. Listing 3).

**Listing 1.** Sleep segment Microsoft.
segmentType: “Doze”, “Snooze”, “Awake”, “Sleep” sleepType: “Unknown”, “RestlessSleep “, RestfulSleep { “dayId”: “2015-09-17T00:00:00.000+00:00”, “sleepType”: “RestlessSleep”, “segmentId”: NumberLong(635780414429706039), “startTime”: “2015-09-16T23:04:02.970+00:00”, “endTime”: “2015-09-16T23:08:59.970+00:00”, “duration”: “PT4M57S”, “heartRateSummary”: { “period”: “Segment”, “averageHeartRate”: 78, “peakHeartRate”: 85, “lowestHeartRate”: 67}, “segmentType”: “Sleep” }

**Listing 2.** Sleep segments in Fitbit.
1 (“asleep”), 2 (“awake”), or 3 (“really awake”) [{ “dateTime”: “23:38:00”, “value”: “1” }, …]

**Listing 3.** Sleep segments in Jawbone.
1 (awake), 2 (light) or 3 (deep) [ { “depth”: 1, “time”: 1442278000 }, …]

### 4.3. Temporal Discrepancies

In addition to the variety of data models, differences can also be found in the identification of the traces. In the case of the sleep periods, it is important to identify the concrete day at which such a period is produced. This can be seen as a trivial task, but different vendors attribute it in different ways:

- Microsoft identifies different sleep periods using a single identifier for each day. Therefore, if a user sleeps from day 6 to day 7, that sleep period is assigned to day 6. If the user sleeps after midnight, the sleep period is assigned to the next day. As a result, the data can show weird things: some days the user does not sleep at all, while other days he/she sleeps almost all the day.
- Fitbit identifies each sleep period using a different identifier. For each day, one of the periods is marked as the main one and the other periods, if available, as naps. If a user sleeps from day 6 to day 7, the sleep period is assigned to day 7.
- Jawbone follows an approach similar to Microsoft. Nevertheless, if a user sleeps from day 6 to day 7, the sleep period is assigned to day 7.

### 4.4. Differences in Counters

Different vendors follow different approaches to count events produced. More precisely, they vary the way in which counters are reset. Microsoft Band counts all the items (e.g., number of steps, calories or distance) produced from the last formatting of the device. On the contrary, Android Wear devices reset their counters when the clock of the device is rebooted. In a different way, Fitbit counters are reset every day. Obviously, these differences need to be taken into account if data from different sensors need to be integrated or compared.

## 5. Use Cases

Our research group has focused on the development of Learning Analytics solutions. A main goal has been the development of a server using software tools (e.g., Jersey [53] for the server API, Weka library [54] for analytical calculations, etc.) to support different learning strategies, classroom management, recommendations, etc. In this context, wearables have been identified as an opportunity to collect student data and analyze it to provide indicators, such as the following ones related to stress and sleep [14,15]:

**Sleep Quality (SQ).** This indicator is based on the well-known Pittsburgh Sleep Quality Index (PSQI) [55]. The idea is to obtain a daily measure of how well a person sleeps, providing a value between 0 and 100. Our calculation method, based on machine learning classifiers, is flexible, not as rigid as the original method to calculate this index. Particularly, we use a classifier algorithm (Kstar) trained with the daily sleep data and the answer provided by the person about his rest feeling. This training data is captured during a 30-day period. The factors used to perform the calculation are: sleep duration, fall asleep, awake, HR min in the night, average skin temperature and quiz answer. During the first 3 days, the classifier will not provide any value. After this initial period, the system provides an estimation of the sleep quality. This estimation improves along the following days when more data is provided. After 30 days, the user is not required to answer the question about his rest feeling and the system provides the SQ indicator in an automatic way.

**Sleepiness Level (S).** This indicator provides the somnolence or activation level of a person in real time. To estimate this level, we used machine learning techniques over a large number of appropriately tagged tracking records related to the movement and sleep of the person. The following factors are used: accelerometer, heart rate (HR), skin temperature (ST) and sleepiness type. To train the model, we use two methodologies. The first one collects HR, ST and accelerometer data during a short time period (10 min) at midnight. These samples are related to a somnolence level. The second one involves the use of an app that queries the user about his somnolence or activation level. We decided to use the J48 classifier (commonly known as C4.5) of the Weka library because it is fast and simple and when tested against other classifiers, it has offered good reliability results. After the initial training, the server processes the samples collected from the users and estimates sleepiness levels continuously.

**Chronotype.** This indicator seeks to identify the student chronotype. To calculate this indicator, we use sleep data distinguishing between working (studying) days and non-working days (holidays, weekends). We assign a specific score to each measure using the hour/score equivalence table of the morningness-eveningness tests [56,57]. A score between −2 and 2 is assigned depending on the hour at which the person goes to sleep (start bedtime) and wakes up (end bedtime) as we can see in Table 3. The result is a value between −2 (definitively evening) and 2 (extreme morning).

**Stress.** This indicator provides a mean of the stress level of the student. To calculate it, we follow a methodology similar to the one used to calculate the somnolence indicator, using the J48 classifier as a predictor. We reviewed the literature to search for biometric data used in the calculation of the stress and found the next ones as the most commonly used [58,59,60,61]: heart rate (HR), pupil diameter, blood volume pressure, skin temperature (ST) and galvanic skin response (GSR). From the available wearables and smartphones, it is not possible to measure neither the pupil diameter nor the blood volume pressure. Therefore, we used the HR, ST and GSR. Nevertheless, the GSR is also difficult to obtain and we use the accelerometer to provide a measure of the user activity level in case GSR is not available. Another factor was included, Stress Type, to allow the user to indicate his stress level and train the model.

To provide these indicators we considered the use of different wearables as data sources. The calculation of the sleep indicators involves some requirements related to the availability of the data. In the case of the Sleep Quality and Chronotype, we do not plan to offer this data all the time, but at some specific time points. This can be done at the end of the day, for example. To do so, we can obtain data provided by proprietary warehouses. On the contrary, the Sleepiness or Stress level needs to be calculated constantly to detect when drowsiness is produced and notify this to the user (teacher or learner). Therefore, we need to collect data in real time.

The proposed indicators can be useful to support learning scenarios, such as the development of Self-Regulated Learning strategies [62] or pedagogical development by following Action Research approaches [63]. In Self-Regulated Learning, learners are asked to self-monitor and reflect on their activities and performance. For example, they should know what activities are stressful or boring for them. In Action Research, teachers are asked to register observations of their instructional activities and to reflect on them. Teachers can use the proposed indicators to discover the state of their learners at different hours or their stress during different activities.

Next are described some of the usage scenarios we are working on:

- Personalized work plan. Taking into account the chronotype, the time periods with more sleepiness and stress level, a working plan will be generated suggesting studying, physical activity and relaxing hours. Learning and training activities will be assigned to the periods at which the user is more active.
- Student working groups. Students are grouped taking into account their chronotype. Students with a similar chronotype and somnolence periods will be grouped together.
- Recommendations. The introduced indicators made up a learner profile. This will enable us to apply clustering algorithms to identify similar learners and provide recommendations based on neighbourhood. For example, to perform similar activities or adopt the study routine of successful learners.
- Alerts. These can be generated when anomalies are detected in relation to the sleep and stress patterns captured in the indicators.
- Self-quantification. The values and indicators will be shown to the student (and teacher) on a specific dashboard. Daily, weekly and monthly summaries together with comparisons with other students will be provided. This can be used as a tool to promote self-reflection and self-regulation, similar to what is already done in sport activities. It will enable the user to be more aware of his own features and patterns.
- More information will be provided to the teachers about the student activities related to their subjects. Particularly, classroom measures of the stress or sleepiness levels during different academic activities can be provided. This will enable teachers to plan their teaching, taking advantage of the periods at which the students are more activated. Similarly, activation and relaxing activities could be introduced to reduce the sleepiness and stress at certain situations.

### 5.1. Selected Devices

As a first stage in our piece of research, we faced the “device fragmentation” problem. We took into account the following criteria to select the wearables to be used in our experiments:

- Developer’s market share. It is important to use common devices in the market because we are mainly interested in their availability for our experiments.
- The availability of sensors. We are working on the development of algorithms that do not require many sensors, but some of them are key in order to calculate the indicators. Therefore, a minimum set of sensors should be available: accelerometer and HR in most cases are necessary.
- The options to collect data from the device. We pay attention to the availability of some API or method that enables us to collect data from the device.
- Price and availability. Every student needs to obtain his/her own wearable. Therefore, from a sustainability point of view, we tried to work with devices that were easy to acquire and affordable.

Based on these criteria, we considered it would be a good option to choose Fitbit, Xiaomi, Apple and some Android Wear devices to work with Google Fit. However, Xiaomi was discarded because, despite it having a significant market share, it does not provide any method to gain access to wearable data. We also discarded the Apple iWatch because of its high price and proprietary platform that creates difficulties to transfer data to our system. In addition, we also were interested in using a device with a good number of sensors that could be accessed using different methods. In this way, the Microsoft Band was chosen as a good candidate. As a result, we performed our experiments using the following devices:

- Fitbit Surge. Fitbit had the largest smartband market share in recent years. In addition, it provides a REST API that facilitates data collection.
- LG Watch R. This was selected as a kind of Android Wear device. This device enables access to the sensor data through native apps, but it also supports the Android app and the Web Service provided by Google Fit.
- Microsoft Band. This was selected taking into account the relevance of the company supporting it. In addition, it includes a good set of sensors and provides easy access to data through its API.
- Jawbone. This device provides sleep information based on a unique sensor: an accelerometer. It was selected because it is a very affordable device and the developer is well-known in the wearable market.

#### 5.1.1. Fitbit Surge

This wearable is one of the best smartbands for sport available. It is based on a proprietary software framework, not a real OS, and in this way it is not possible to execute any app on it.

Fitbit has some special features that make it an interesting device. It can store data over several days without loss because its energy needs are very low. The problem is that, after a certain level of storage is achieved, it performs a summarization and raw data is discarded. This can be considered as a disadvantage, but also as a positive feature because the device keeps the more relevant information by itself.

This wearable includes a good number of sensors as is shown in Table 4. The problem is that data collected is translated into variables focused on sport and fitness features, such as calories consumed, steps walked, altitude, or distance; not raw data from the accelerometer.

**Figure 9:**
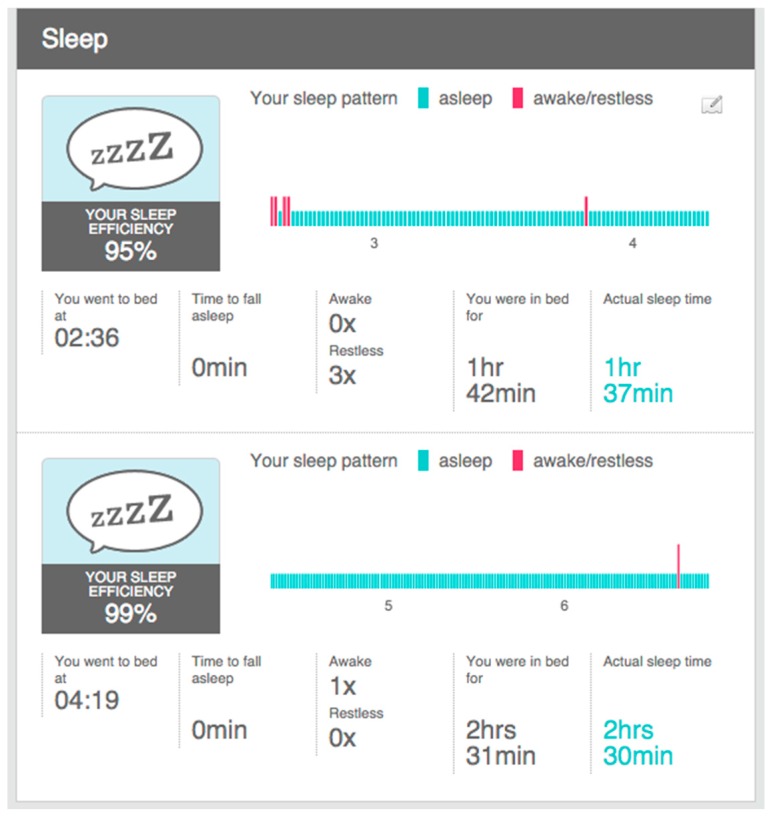
Sleep analysis by Fitbit based on data from the Fitbit Surge.

From the point of view of our piece of research, it provides many interesting sleep features, (cf. Figure 9): identification of sleep periods, the number of awakes, time to fall asleep, total time, and real time.

#### 5.1.2. LG Watch R

The LG Watch R is based on the Android Wear OS. As a result, it is compatible with any Android device, particularly smartphones. To enable the connection between both devices, the Android Wear app has to be installed in the Android device.

This wearable does not include a large number of sensors as is shown in Table 4. In addition, to collect the HR data along time, a specific app needs to be developed and the precision is fairly bad, mainly if the device is not well suited to the wrist, or if the user is moving. Surprisingly, if the device is not being worn on the wrist, sometimes it shows a constant HR value. The use of the HR drains the battery quickly and it has a bad precision.

This wearable does not provide any type of analytics for sleep. Therefore, for our research we need to perform all the analytics related to the identification of start and end of sleep, movements, etc.

#### 5.1.3. Microsoft Band

This wearable is based on a proprietary OS provided by Microsoft. It can be connected to almost any smartphone (Android, iOS, Microsoft) and to any Microsoft Windows computer.

In addition to the sensors included, it provides some additional functionality, such as notifications and calendar to facilitate the user’s access to smartphone services. The problem is that this functionality drains the battery quickly. In this way, this device is a cross between a smartband and a smartwatch.

**Figure 10:**
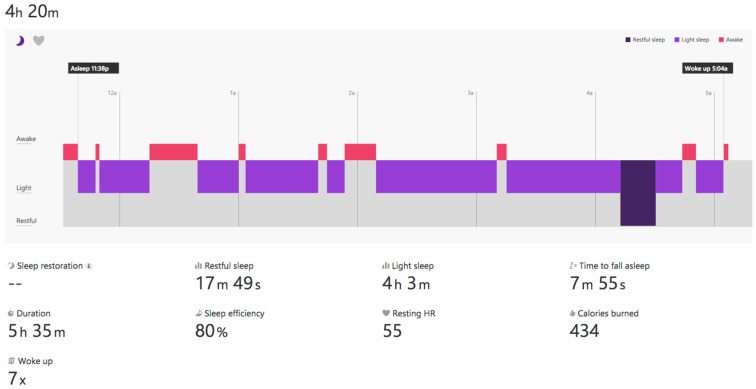
Sleep analysis by Microsoft through the Microsoft Band.

This wearable is one of the most complete in the market as is shown in Table 4. The Microsoft proprietary warehouse performs some analytics and provides some specific indicators, such as sleep features (cf. Figure 10). Nevertheless, these features cannot be automatically related to the data directly extracted from the sensors. The problem is that the user identification in the wearable is not related to the user identification in the Microsoft warehouse. In the case of our students, we need to instruct them to register in the warehouse with the same e-mail address as in the wearable.

#### 5.1.4. Jawbone UP Move

This wearable is very basic and simple. Its framework is focused on reducing the energy consumption. As a result, its non-rechargeable battery has a duration of 6 months approximately.

Jawbone has its own proprietary warehouse and specific Android and iOS apps to perform the data transfer. Nevertheless, it is not possible to develop native apps for smartphones. An SDK for the development of Android apps is provided, but just to facilitate processes related to the authentication and to the recovery of data leaks in the Jawbone proprietary warehouse.

The simplicity of this wearable is clear as is shown in Table 4. As in the Fitbit case, the data taken from this sensor is processed to provide many different kinds of data: number of steps, sleep indicators, etc. The same developer has another wearable of a higher category, Jawbone 3, which includes much more sensors: HR, breathing and GSR.

**Figure 11:**
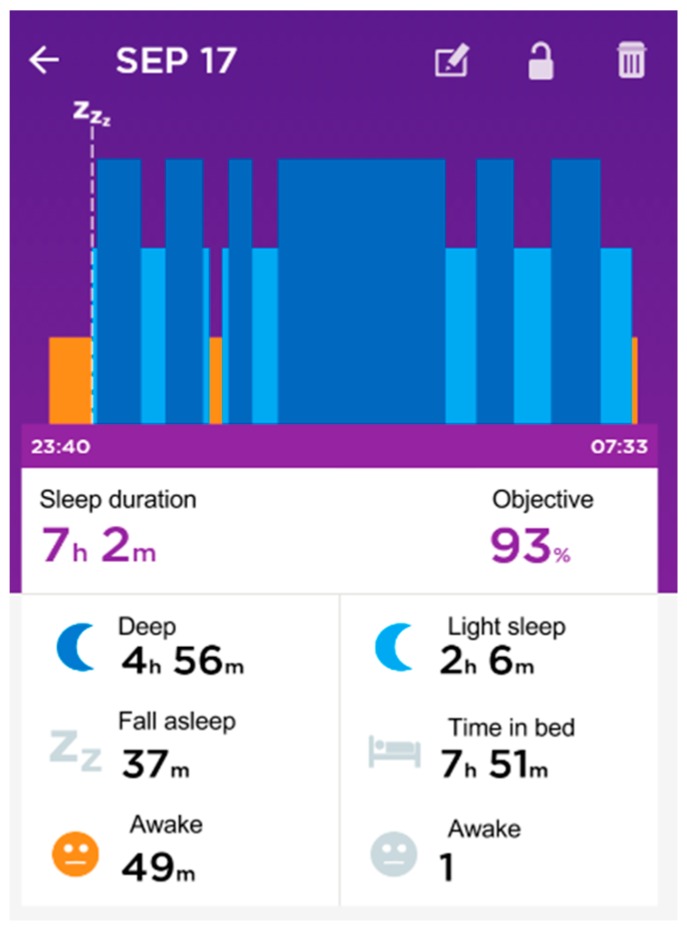
Sleep analysis by Jawbone through the Jawbone Up move.

There are some issues to note related to the sleep indicators, (cf. Figure 11). The wearable does not capture the sleep start in an automatic way. The user is required to push a button in the device to indicate the start. This is used to change between rest and physical activity phases. Alternatively, using a smartphone app the user can add the sleep period to the warehouse.

#### 5.1.5. Summary

In Table 4 we can show a summary of the sensors available in the devices under study.

### 5.2. Data Transfer

**Figure 12:**
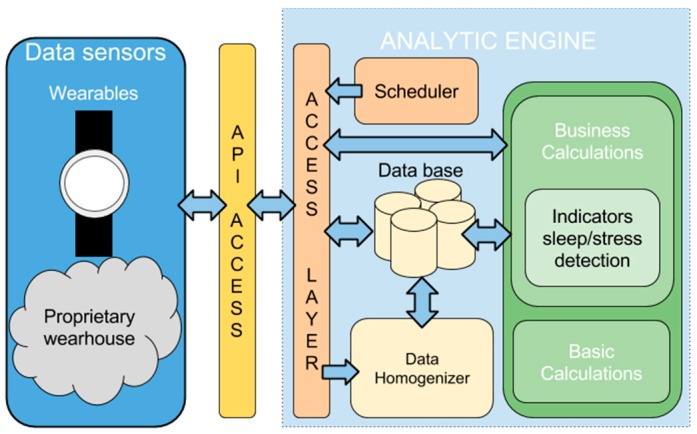
Architecture of our analytic engine.

As it is explained in Section 3.2, there are different ways in which wearable sensors’ data can be transferred to our server. In this section, we explain the options adopted in the selected devices. A software architecture has been developed to perform this transfer and process the data towards the desired indicators, (cf. Figure 12). This architecture involves several modules, each one with a specific function that is described in the next sections.

#### 5.2.1. Fitbit Surge

This wearable allows the warehouse data transfer—direct access. To initiate the transfer and synchronize the data, a Fitbit specific app needs to be installed in the student’s smartphone (Android, iOS or Windows) or computer (Windows). This enables the data transfer from the wearable to the Fitbit proprietary warehouse.

The data available to third parties in the Fitbit proprietary warehouse is about physical activities: walked distance, consumed calories, steps number, time devoted to each exercise, etc. HR over time and sleep features are also provided. Due to privacy issues, in order to collect some special data, such as the minute-by-minute HR, we need to contact the company.

#### 5.2.2. LG Watch R

The LG Watch R enables the data transfer in accordance to the following modes:

- **Wearable data transfer—indirect access**. If we use the Google Fit SDK, we can gain access to the wearable sensors to obtain HR and steps, but we cannot obtain accelerometer or gyroscope raw data. To retrieve such data, we would need to develop and install native apps both in the wearable and in the smartphone. Using this mode, the following data elements can be collected: HR, accelerometer, xyz position, atmospheric pressure, steps, calories and distance. In this case the data can be collected in real time.
- **Warehouse data transfer—direct/indirect access.** The wearable sensors’ data is transferred through the smartphone to the Google Fit warehouse. Then, warehouse data is requested by our server on a daily basis. The data collected in this mode is limited: distance walking/by bike/running, calories consumed, number of steps, time spent in each type of exercise, and if the user has manually taken his/her HR, this value is also included.

#### 5.2.3. Microsoft Band

Microsoft Band supports the two data transfer modes:

- **Wearable data transfer—indirect access**. The Microsoft Health app needs to be installed in the wearable and a native app has to be installed on the smartphone. Microsoft provides a library set (SDK) compatible with Android, iOS and Windows that can be used to implement the smartphone app. Using this mode, the sensors’ data can be collected in real time. Nevertheless, not all the data from the available sensors can be collected. For example: light sensor, GPS and galvanic sensor. In addition, it is important to note that in case the connection is broken, the data about HR and skin temperature is lost. Another issue to have in mind is that some data, such as the number of steps and calories consumed, is provided as a number since the beginning of time. This means that the number of steps in a certain day has to be computed as the difference between the value at the end and at the beginning of the day.
- **Warehouse data transfer—direct access**. The sensors’ data stored in the Microsoft warehouse is summarized and processed in accordance to the Microsoft models. As a result, the data available for third-parties is: distance, calories consumed, number of steps, time devoted to different exercises, HR according to the activity type, sleep analysis, etc. In the case of sleep analysis, it provides both the sleep stages and the HR in time intervals (cf. Figure 10).

#### 5.2.4. Jawbone UP Move

This device just supports the warehouse data transfer—direct/indirect access. To perform the data transfer from the wearable to the proprietary warehouse a Jawbone app needs to be installed on a smartphone. The warehouse provides data related to the physical activities of the user (distance walked, calories consumed, number of steps, time devoted to each exercise) and sleep analysis. Nevertheless, it is not possible to collect the nominal values from its sensor.

#### 5.2.5. Summary

As a final reflection, it is important to note that current wearables feature quite limited capabilities that restrict their performance. One of the most important limitations is memory size. As a result, the number of data they can store is rather reduced and frequent data transfers are needed. Next, issues need to be considered related to this limitation:

- What happens if the (Bluetooth) connection to the intermediate system (e.g., smartphone) used to transfer the data to the proprietary warehouse or to the third-party server is broken? In the Microsoft Band case, when the connection between the wearable and the smartphone is broken, the sampling rate of sensors’ data is reduced to save memory. When the connection is recovered the saved data is sent to the intermediate system as soon as possible.
- What happens if the connection between the intermediate system and the warehouse/server is broken? In this case, the data is stored in the intermediate system because it has a much larger memory capacity than the wearable. The situation in which this memory is completed is not considered.
- What happens if the wearable is not able to transfer the data and is running out of memory? Usually, as for Fitbit, if the wearable is running out of memory it summarizes the data collected and in the worst cases, it removes the older summaries.
- How is the permission to obtain users’ private data from the proprietary warehouse granted? To receive permission to access data stored in warehouses, such as Fitbit or Google Fit, methods available in the REST API or SDKs need to be invoked on an individual basis. Using a specific web page, the user will check the permission requests and will confirm them to the third-party. Once a permission is granted a “token” is provided that can be used to collect the private user data from the warehouse.
- How long is the duration of the token? The token has a limited valid time because of security concerns. Once the user has been granted permission to gain access to his/her data, a “refresh token” is also provided, in addition to the “token”. When the lifetime of the “token” expires, the “refresh token” can be used to generate a new “token”. Currently, there are great differences in the duration of the tokens for different vendors, while in some cases they are in the order of hours, in other cases they are in the order of years.

Table 5 summarizes features related to the data transfer and battery duration.

### 5.3. Integrating Data

Using the available transfer modes, the selected wearables provide us the data to calculate the sleep and stress indicators. Nevertheless, before data analytics can be performed, the differences among data coming from devices of different vendors need to be tackled.

#### 5.3.1. Architecture

The architecture of our Analytic Engine shown in Figure 12 is in charge of the homogenization, storage and analytics of data. The architecture is made up of several modules. The first one is the task Scheduler in charge of the programmed tasks such as the data collection in proprietary warehouses and the periodic calculation of sleep and stress indicators. Another module is the Access Layer, which is used as an entry point of the data sent by smartphones and PCs or collected from proprietary warehouses. Once the data is in the Analytic Engine, the Data Homogenizer module prepares and stores the data to enable its processing in a homogeneous way. The last modules of the architecture, located on the right side of the figure, involve the data analytics. A Basic Calculation module is included to perform basic operations, such as correlation calculations and statistics calculations. The Business Calculation module involves machine learning techniques to detect routines and outliers.

Results obtained from the analytics are stored in the database. This data is provided to final users using a dashboard, as part of recommendations, etc. To support this communication a REST API is included enabling the receiving and sending of information. In case of real time services, the data is provided directly from the calculation modules.

During the development of this architecture we have taken the following decisions:

- All the wearables that enable the transfer of data in real time to our server follow the same data model. This model is shown in Listing 4.
- All the data objects include a unique id, the learner id, the date as a String and as a Long integer, the learner id in the proprietary warehouse and the device id. This data is shown in Listing 5.
- The sleep data analysed every day is collected from the source and prepared in accordance to the model shown in Listing 6.

**Listing 4.** Real time data object of a wearable.
**private** String device; **private** Long date; **private**
**int** wear; **private**
**int** resistence; **private** String uvValue, typeWalk; **private**
**double** tempValue, speedValue, disValue, accelValue; **private**
**long** stepsValue, caloriesValue, hrValue; **private** String typeStudy;

**Listing 5.** ID’s data object of a wearable.
**protected** String _id; **protected** String student_id; **protected** Long date; **protected** String student_id_api; **protected** String device;

**Listing 6.** Homogenized sleep data
{ “_id”: “—”, “student_id”: “—”, “date_string”: “2015-07-22”, “date”: NumberLong(1437523200000), “student_id_api”: “—”, “device”: “Microsoft_Api”, “data”: { “total_time_data”: 394, “time_to_sleep_data”: 11, “temp_mean”: 34.56, “hr_min”: 56, “num_awake”: 9, “awake”: 86, “light”: 221, “relaxed”: 87, “sleep_efficiency”: 80, “efficiency_proposal”: 70, “start_hour”: “01:26:18”, “end_hour”: “08:00:48”, “total_time”: “06:34:30”, “time_to_sleep”: “00:11:24” }, “list”: { “awake”: [1,0…], “light”: [0,1,…], “relaxed”: [0,0,…], “time”: [“23012618384”,”23013200000”,…], “hr”: [64, 64,…], } }

From a more technical point of view, our server is made up of the following components and libraries: Java 8, Tomcat 8 as a server, Jersey [53] to generate the REST API and MongoDB as a non-relational database. Java 8 is the most recent version of Java, fully compatible with previous versions. A key point in this version is the support of Lambda expressions, that facilitate the management of lists using map/reduce filter functions over data streams. Also, several statistical and machine learning libraries are used to calculate the indicators:

- Apache Commons-math3. Library with statistical functions such as: standard deviation, linear regressions, matrix operations, etc.
- Weka [54]. This library is used to solve machine learning developments. The classifiers allow us to provide real time predictions for the indicators: stress, sleepiness, etc.

**Figure 13:**
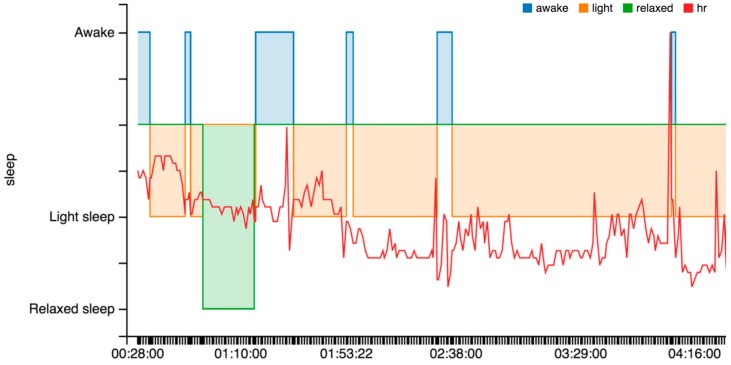
Sleep segments in Microsoft.

**Figure 14:**
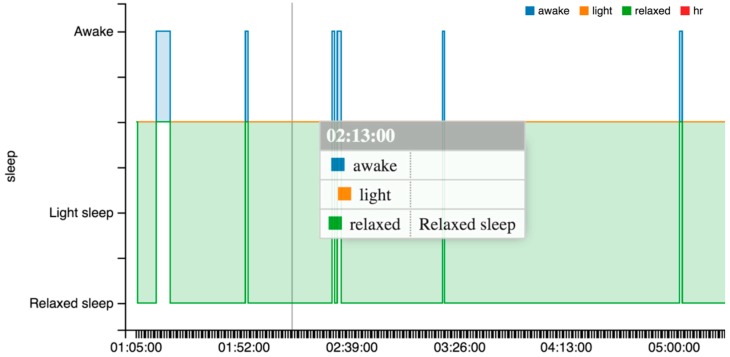
Sleep segments in Fitbit. We only can detect two states of sleep in Fitbit wearables.

**Figure 15:**
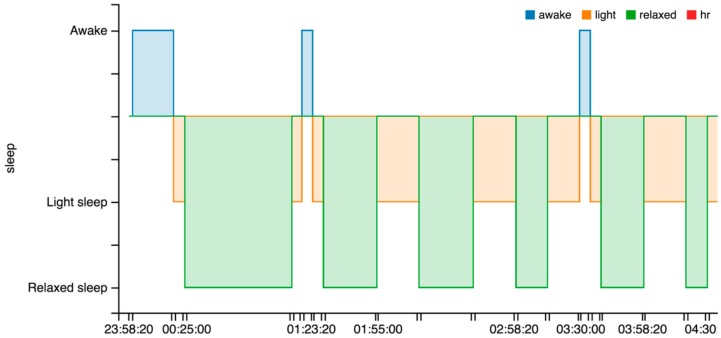
Sleep segments in Jawbone.

The server involves several classes related to the homogenization of data, statistics, predictions and indicators. These classes are orchestrated to carry out the following data flows:

- Input data flow. This is related to the collection of data from wearables, data homogenization and storage in the database. It can be produced in two different ways:
  - OnDemand. This is related to the “indirect access” mode. The intermediate device needs to include the software that collects data in the wearable or in the warehouse and send it to the server through POST requests. Next, calculations included in the classes StatisticsCalcs, WekaCalcs and IndicatorsCalcs are applied to get the indicators.
  - Scheduled. Every morning at the same time (12:30 p.m.) the server performs the update of tokens and initiates the collection of data from the registered users. GET or POST requests are used in accordance to the different options.
- Output data flow. A class based on the Jersey library implements GET methods to provide homogenized data to the external services. This data can be visualized in dashboards, similar to the graphics shown in Figure 13, Figure 14 and Figure 15, to generate notifications or recommendations.

Finally, we show the structure or our MongoDB database. Mongo is organized in Collections, equivalent to tables in a SQL (Structured Query Language) data base systems. The following collections are included:

- Proprietary systems collections. We store data collected from proprietary systems in sleep_fitbit, sleep_microsoft, sleep_jawbone, etc. We also maintain the tokens needed to carry out the scheduled daily data collections in fitbit_token, microsoft_token, jawbone_token, etc.
- sleep_analytics. This collection stores the homogenized sleep data per user, device and date. It maintains no-real time indicators in accordance to the data model shown in Listing 6: sleep quality and chronotype.
- band_sensors. This collection stores the sensors homogenized data. Each entry corresponds to a specific user, device and date. The data is arranged in accordance to the model shown in Listing 4.
- sleep_quiz. This collection stores the answers of the users to the sleep questionnaire. Each entry is related to a specific user and date.

#### 5.3.2. Differences in Data Models/Names

We need to get the information about sleep segments following the same model. It is possible to transform the Fitbit and Jawbone models to the Microsoft in the following ways:

- Fitbit has a main problem because there is not a one to one mapping between Fitbit (“asleep”, “awake” and “really awake”) and Microsoft (“awake”, “light” and “deep”) sleep types. In Fitbit, the “awake” state is assigned to very short periods produced when the user is moving while sleeping. Therefore, we decided to assign “really awake” and “awake” the value “awake”. As a result, we just used “asleep” and “awake” types.
- In the Jawbone case, we need to use two records to create a sleep segment. We use the start time of both of them to indicate the start and finish period.

The result of a visualization taking into account these indications is shown in Figure 13, Figure 14 and Figure 15. The data collected from the three different proprietary services is adapted for processing in our analysis service. A sample of the object produced with the segments homogenized is shown in Listing 6 in the previous section.

#### 5.3.3. Temporal Discrepancies

We decided to adopt the Microsoft identification, but marking the longer sleep period as the main one and the other periods as naps. The sleep period if a user sleeps from day 6 to day 7 is assigned to day 6, even if the user begins to sleep after midnight.

#### 5.3.4. Differences on Counters

Many systems usually provide daily summaries of different variables. To do this, in the Microsoft case we compute the differences between the values at the beginning and at the end of the day. In the Android Wear case, we developed a strategy based on clock data. All the changes detected in variables are recorded. When the clock is reinitiated, the last value available for the variables is recovered and it is taken it into account to calculate the daily summaries.

#### 5.3.5. Sensors Standardization

As is shown in Section 2.3, different wearables include different sensors. As an initial approach to manage the lack of some data because the corresponding sensor missed, we use a special value (−1). As a more elaborated approach, we try to get the data from missed sensors looking at some alternative sources. For example, the LG Watch R does not include a GPS sensor, but almost all smartphones provide it. Therefore, the smartphone can be used to collect the GPS data. This approach can be extended to other devices accessible from the smartphone. If a person is using a wrist wearable and a smartphone, data from both devices could be combined as fusion data [64].

#### 5.3.6. Analytics Location

**Figure 16:**
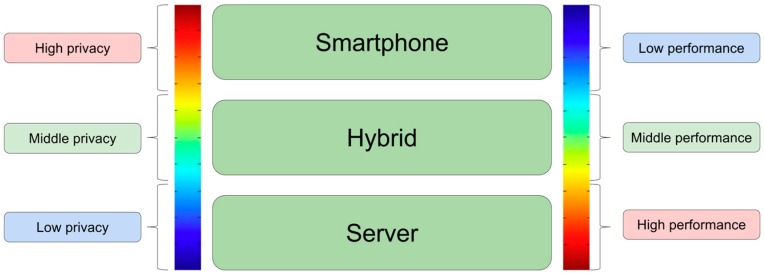
Analytics location models.

We consider several options to perform the processing of the analytics algorithms in accordance to the different systems included in the data transfer option. Initially, such processing is done at the server, but in the wearable data transfer it is also possible to perform some operations at the intermediate smartphone. For example, the average value of the HR in a time period can be calculated either in the server or in the smartphone. Therefore, three different scenarios to perform the analytics are identified (cf. Figure 16):

- All the analytic processing is performed in the smartphone. In this case, the smartphone transfers to the server just the results obtained from the analytics, not the raw sensors’ data. This can be a valuable advantage from privacy and data protection points of view, because the data shared is not so sensitive. For example, accelerometer and HR data can be used to get many different results, more than if just the sleep features are available. In addition, the data transfer load is highly reduced. On the contrary, the performance of analytics in the smartphone is very low and drains the battery. This option is not feasible if wearable data transfer from the wearable to the smartphone is not feasible.
- All the analytics processing is performed in the server. The advantage of this solution is that algorithms can be changed easily and for all the devices involved at the same time. There would be just one implementation for each algorithm, and not several versions such in the smartphone case where multiple implementations would be required for different developers. In addition, the performance of servers is much better than the performance of smartphones. In the smartphone case, the analytics should be performed in the background.
- In a hybrid approach, processing in the smartphone and in the server, the algorithms are split between both systems trying to get the better performance and to ensure data privacy issues. Particularly, the homogenization operations can be performed in the smartphone.

## 6. Standardization Context

The work described in this paper is about the processing of data collected from wearable sensors and transferred to servers. Similar works with different goals but using the same approach can be found in the recent literature. In [65], an architecture is proposed involving a smartphone to transfer data from a sensor to a server to calculate insomnia indicators. The key difference with the solution described in this paper is in the type of devices. In our case, we use commercial devices from different developers and the provided communication protocols. Android Wear and Google Fit, as described in Section 4.4, have been proposed as solutions to standardize data collection, but they are not broadly supported by commercial products; and other issues, such as homogenization, need to be considered.

From a technical point of view, the work described in this paper is about data communication and homogenization, involving different types of devices: wearables, smartphones, computers and servers. This is situated in the current context of the Internet of Things (IoT) and Machine to Machine (M2M) standardization. There is a need for interoperability between different devices that can behave more or less as autonomous entities in an ecosystem. Communication is a main challenge, but other issues also are critical: security, privacy, trust, etc. In this section we review the current work in this area.

### 6.1. Key Concepts

The IoT vision involves the connection of all kinds of physical devices to the virtual world, supporting their communication with existing Internet entities. No longer just people or software systems would exist in the virtual world, but also a myriad of devices that could be addressed, identified, located, sensed, actuated and, in general, interacted using information and communication technologies [19].

It is expected that the IoT has a strong impact on many aspects of human daily file in the near future. There is a common understanding that new technologies will soon enable the inclusion of communication modules, microprocessors and all kinds of electronic components as miniature pieces into everyday objects, making such objects smart entities. As a consequence, there will be a radical change from the 99% of things not connected to the Internet today to the 37 billion new things connected by 2020 [66].

M2M is part of the vision of the IoT [20]. Machines are considered in a broad sense as any type of device: computers, mobile devices, wearables, robots, cards, etc. The communication among machines can be performed in different ways using wireless or wired networks. Eventually, the M2M communications will allow end-users to get data about events of machines [67]. M2M communications involve three phases: collection of data from sensors, transmission of the collected data to external systems and processing of data to provide some kind of result.

### 6.2. Initiatives

Standards provide many benefits. Products developed by different vendors following standards are compatible and can work together. Furthermore, standards ensure the quality and sustainability of products. In the ICT and electronics industry there already exist many standards. Nevertheless, the needs and requirements involved in the development of the IoT and M2M concepts demand the development of new solutions and models. In this way, in recent years, main standardization bodies have launched several initiatives towards the standardization of the IoT and M2M domains:

- The ISO/IEC JTC1 established the Special Working Group (SWG) 5 on the IoT in 2012 to identify gaps and market requirements related to the IoT. In November 2014 JTC1 decided to establish a new Working Group WG 10 on the IoT to create standards, coordinating the standardization work and cooperation with other organizations. Currently, this group is working in the ISO/IEC 30141—the IoT Reference Architecture but it is just at a preparatory stage.
- The “oneM2M—Standards for M2M and the Internet of Things” is a partnership of main standardization bodies, such as the European Telecommunications Standards Institute (ETSI), the US Alliance for Telecommunications Industry Solutions (ATIS) and Telecommunications Industry Association (TIA) and similar bodies in Japan, China, India and South Korea. It is also made up of 232 members from main electronic, computer and communication vendors. It is focused on the standardization of end-to-end M2M communications [25]. The OneM2M Technical Specification published in January 2015 specifies the functional architecture for the Services Platform, but it is still under development [26].
- The IEEE Standards Association has launched the project P2413—Standard for an Architectural Framework for the IoT to promote cross-domain interaction, aid system interoperability and functional compatibility in for the IoT [24]. More related to the contents of this paper, the IEEE has also launched the P360—Standard for Wearable Consumer Electronic Devices—Overview and Architecture. Its main goal is to provide a testing solution and a homogeneous way to describe the features for wearable consumer electronic devices. Currently, both proposals are under development.

These initiatives show a clear interest for the standardization in the IoT and M2M domains. Nevertheless, it is a process under development. Many standards for specific issues are already available and are being generally adopted, such as standards for near field wireless communication. Bluetooth is the most widely adopted technology, but other options such as ZigBee, WiFi or ANT are also available [68]. Other specific proposals are also quite advanced, such as web protocols as protocol bindings for HTTP (Hypertext Transfer Protocol) and Constrained Application Protocol (CoAP).

**Figure 17:**
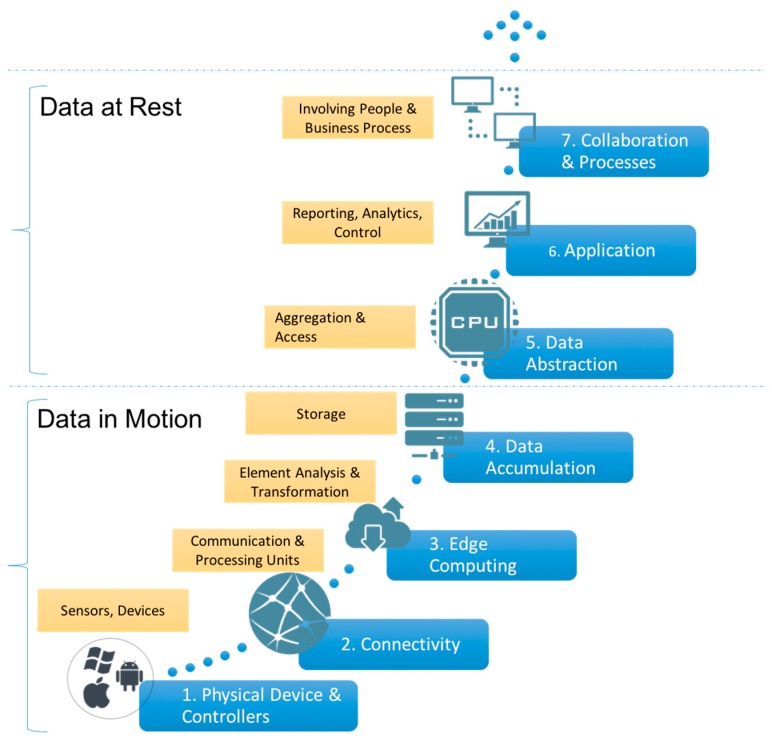
Adapted representation of the IoT Reference Model by Cisco System, Inc. [66].

A main interest for the standardization bodies is the development of a framework or reference architecture that arranges the different issues involved. The three aforementioned initiatives are working towards such a result, but currently there is no clear proposal from any of them. Cisco Systems, Inc. has published a white paper with a particular IoT Reference Model that deserves special attention [66]. It is made up of seven layers (cf. Figure 17): (1) Physical Devices & Controllers where sensors and actuators are situated; (2) Connectivity involving reliable and timely data transmission; (3) Edge (Fog) Computing involving initial data element analysis and transformation; (4) Data Accumulation to store data permanently; (5) Data Abstraction, providing services to aggregate and store data; (6) Application, related to the development of reporting, analytics and control; and (7) Collaboration & Processes, involving people and business processes. This model groups layers under two different categories. Layers 1 to 4 are named as “Data in Motion” because data is processed and transformed while it is transferred from lower layers to upper layers. Layers 5 to 7 are named as “Data at Rest” because data is unchanging and stored in memory at each layer. Layer 3 is specifically tagged as “fog computing” because it refers to the idea that information processing can be initiated as early and as close to the edge of the network as possible. In this way the payload of the communications and processing in upper layers is reduced.

## 7. Conclusions

This paper analyses the issues involved in the collection and homogenization of data from wearables of different providers. The problem is about systems’ interoperability and data integration. In this way, it can be considered as a standardization problem related to the IoT and M2M domains. The paper contributes to identifying key issues that need to be considered to support interoperability in this domain, such as wearable devices fragmentation, differences in options to gain access and transfer data both from wearables and from warehouses, differences among the data models, etc. Platforms promoted by main vendors (Google, Apple and Samsung) have made attempts towards the unification of the data collection from different devices. Nevertheless, from the analysis, it is shown that great differences still exist among the options available. In addition, to date, none of these solutions have been adopted by the community as a whole and just some devices of other vendors have been integrated at each platform. It is also important to note that other vendors exist, such as Fitbit, Microsoft, and Jawbone, that have focused on supporting their own wrist wearables, without any concern about the integration of data from other vendors.

The need for standardized data models and data transfer solutions in the wearable domain is clear from the point of view of our piece of research. Data provided by wearables is oriented towards being stored over time, compared with data from the same and other users and processed applying data analytics and machine learning techniques. In a general context, as in our educational scenario, different users will use different devices and, moreover, they will change wearables quite often. We have focused the second part of the paper on introducing the work we are carrying out in our heterogeneous and multiple-user scenario where this situation is clear. In this scenario, we have shown the great potential of data provided by wearables to calculate interesting features for students, particularly sleep and stress indicators. These features can be used to support both teachers and learners in many different ways. Nowadays, issues related to the motivation, engagement and healthy habits are considered very relevant and wearables can provide important data to report on them. Nevertheless, in order to enable a good treatment and processing of data, the procedures to collect and process it should be clear and standardized as far as possible.

Taking into account our experience, we consider that a multi-layer approach must be adopted towards the development of interoperability solutions in the wearable domain, providing several levels of integration that should be clearly defined. This approach should take into account the following levels: (i) a connectivity level, specifying the technology used to connect with wearables and the method to gain access to data; (ii) a data level, with the definition of homogeneous data models; (iii) an authentication level, related to the permission needed to gain access, the features and duration of tokens; and (iv) finally, a user level providing a kind of user profile, including basic information and indicators such as stress, sleep, etc. In this way, we could develop systems making use of wearables from different vendors and compare profiles of different users. Main standardization bodies in the domain have launched some initiatives, but as it is indicated the issues that need to be tackled need to be extended. This is the case for the Bluetooth standard, where different levels are considered and application profiles are specified to support specific interoperability issues, such as the Hands-Free Profile (HF) to place and receive calls from a hand-free device of the Heart Rate Profile (HRP). Similarly, profiles related to the wearable sensors could be established.

To finish, we would like to indicate that it is not the purpose of our work to identify a winner wearable among those available in the market, particularly taking into account the precision of sensors. It has not been the purpose of this paper to analyse these features. There have been some pieces of research questioning the use of these devices and their sensors for medical purposes [69]. Nevertheless, for the purposes or our piece of research, we do not consider the precision of sensors as something critical. In addition, the precision or reliability issues do not invalidate the interoperability analysis performed. We are sure that all of these issues will be improved in the near future so wearables can be good enough to be used even for medical purposes.

## Figures and Tables

**Table 1:** Systems and options available in wearable platforms.

Platforms	SDK-Sensors	SDK-Warehouse	REST API
Apple Health	-	X	-
Google fit	X	X	X
S-Health	-	X	-
Fitbit	-	-	X
Jawbone	-	X	X
Microsoft Health	X	-	X

**Table 2:** Data transfer options by platform summary.

Platforms	Wearable Data—Indirect Access	Warehouse Data—Direct Access	Warehouse Data—Indirect Access
Apple Health	-	-	X
Google fit	X	X	X
S-Health	-	-	X
Fitbit	-	X	-
Jawbone	-	X	X
Microsoft Health	X	X	-

**Table 3:** Values used to calculate the chronotype.

Values	Start Bed Time	Values	End Bed Time
2	-/21:30	2	-/05:00
1	21:30/22:45	1	05:00/06:30
0	22:45/00:45	0	06:30/08:30
−1	00:45/02:00	−1	08:30/10:00
−2	02:00/-	−2	10:00/-

**Table 4:** Sensors available in the wearables (Notes 1 it is not possible to gain access to this data).

Sensors	LG G Watch R	Fitbit Surge	Microsoft Band	Jawbone UP Move
HR	X	X	X	-
Accelerometer	X	X	X	X
Pedometer	X	X	X	X
Walking Speed	-	-	X	-
Calories	X	X	X	X
Distance	X	X	X	X
Gyroscope	X	X	X	-
Magnetometer	-	X	-	-
Barometer	X	**-**	-	**-**
Altimeter	-	X	-	-
GPS	-	X	X^1^	-
Ambient Light	-	X	X	-
Thermometer	-	-	X	-
Ultraviolet Light Sensor	-	-	X	-
Galvanometer	-	-	X	-
Microphone	X	-	X	-
Analytics Sleep	-	X	X	X

**Table 5:** Features related to the transfer of data from the wearable.

Type	LG G Watch R	Fitbit Surge	Microsoft Band	Jawbone UP Move
Real Time	X	-	X	-
Warehouse data transfer	X	X	X	X
Token duration	(Unknown)	1 month	1 h	1 year
Wearable data transfer	X	-	X	-
SDK	X	-	X	X *
Battery duration with warehouse data transfer	1 day	4 days	3 days	6 months
Battery duration with wearable data transfer	10 h	-	10 h	-

* This SDK only supports processes related to the authentication and to the recovery of data leaks.
